# Cotton rat immune responses to virus-like particles containing the pre-fusion form of respiratory syncytial virus fusion protein

**DOI:** 10.1186/s12967-015-0705-8

**Published:** 2015-11-05

**Authors:** Lori McGinnes Cullen, Jorge C. G. Blanco, Trudy G. Morrison

**Affiliations:** Department of Microbiology and Physiological Systems, University of Massachusetts Medical School, Worcester, MA 01655 USA; Program in Immunology and Microbiology, University of Massachusetts Medical School, Worcester, MA 01655 USA; Sigmovir Biosystems Inc., Rockville, MD 20850 USA

**Keywords:** Respiratory syncytial virus, Vaccine, Fusion protein, Neutralizing antibody titers, Cotton rats

## Abstract

**Background:**

Virus-like particles (VLPs) based on Newcastle disease virus (NDV) core proteins, M and NP, and containing two chimera proteins, F/F and H/G, composed of the respiratory syncytial virus (RSV) fusion protein (F) and glycoprotein (G) ectodomains fused to the transmembrane and cytoplasmic domains of the NDV F and HN proteins, respectively, stimulate durable, protective anti-RSV neutralizing antibodies in mice. Furthermore, immunization of mice with a VLP containing a F/F chimera protein with modifications previously reported to stabilize the pre-fusion form of the RSV F protein resulted in significantly improved neutralizing antibody titers over VLPs containing the wild type F protein. The goal of this study was to determine if VLPs containing the pre-fusion form of the RSV F protein stimulated protective immune responses in cotton rats, a more RSV permissive animal model than mice.

**Methods:**

Cotton rats were immunized intramuscularly with VLPs containing stabilized pre-fusion F/F chimera protein as well as the H/G chimera protein. The anti-RSV F and RSV G antibody responses were determined by ELISA. Neutralizing antibody titers in sera of immunized animals were determined in plaque reduction assays. Protection of the animals from RSV challenge was assessed. The safety of the VLP vaccine was determined by monitoring lung pathology upon RSV challenge of immunized animals.

**Results:**

The Pre-F/F VLP induced neutralizing titers that were well above minimum levels previously proposed to be required for a successful vaccine and titers significantly higher than those stimulated by RSV infection. In addition, Pre-F/F VLP immunization stimulated higher IgG titers to the soluble pre-fusion F protein than RSV infection. Cotton rats immunized with Pre-F/F VLPs were protected from RSV challenge, and, importantly, the VLP immunization did not result in enhanced respiratory disease upon RSV challenge.

**Conclusions:**

VLPs containing the pre-fusion RSV F protein have characteristics required for a safe, effective RSV vaccine.

## Background

Respiratory syncytial virus (RSV) is a very serious pathogen in several different human populations. The virus is the most significant viral cause of lower respiratory tract infections in infants and young children. Nair et al. estimated that world wide there are annually 34 million acute lower respiratory tract infections due to RSV with 3.8 million infections requiring hospitalization and 160,000–199,000 deaths [1]. Elderly populations are also at risk for serious RSV disease, accounting for 10,000 deaths and 14,000–60,000 hospitalizations per year in the US among individuals greater than 64 years of age [2–4]. RSV infection in immunocompromised populations, particularly stem cell transplant recipients [5] and individuals with cardiopulmonary diseases [6], can be life threatening.

Despite the impact of this virus on human health, no licensed vaccine exists. A major problem for RSV vaccine development has been a lack of understanding of requirements for generation of protective immunity to RSV infection. In contrast to most viral infections, RSV infection does not induce durable protective antibodies in humans. Humans can experience repeated infections caused by the same serotype of RSV many times over years or even within the same season [7, 8]. Similarly vaccine candidates thus far tested have failed to induce high levels of neutralizing antibodies and protection from virus challenge in human trials (reviewed in [9, 10]) although they may be protective in murine models. One important issue has been a lack of understanding of the most effective form of the RSV antigens, particularly the F protein, for stimulating potent human neutralizing antibodies.

The paramyxovirus F protein exists in two very different conformations, a metastable pre-fusion form and a very stable post-fusion form [11, 12]. It is proposed that the protein is initially folded into the pre-fusion conformation. Upon fusion activation at initiation of infection, the molecule refolds through a series of conformational intermediates into a stable post-fusion conformation and, in the process, mediates membrane fusion between the viral membrane and target host cell membranes [11–18]. While the pre-fusion form of F protein should be most effective in stimulating optimally neutralizing antibodies, recent studies have shown that the post-fusion form contains at least some epitopes recognized by neutralizing monoclonal antibodies [16, 17]. Thus, it has been argued that a post-fusion F protein can be used as a vaccine [19] and this form of F protein is now in clinical trials. However, Magro et al. reported that most of the neutralizing antibodies in polyclonal human or rabbit anti-RSV immune sera do not bind to the post-fusion F protein [20] leading them to suggest that the majority of effective human neutralizing antibody binding sites reside on the pre-fusion F protein and not the post-fusion form. Supporting this idea, McLellan and colleagues have defined an antigenic site ϕ not present on the post-fusion form of the protein [18] and showed that monoclonal antibodies specific for this site neutralized RSV at significantly lower concentrations than antibodies specific for sites present on both the pre- and post-fusion forms of the protein. Furthermore, McLellan et al. [21] identified mutations in the F protein ectodomain that stabilized the pre-fusion form of the protein and reported that soluble forms of stabilized pre-fusion of protein, in the presence of adjuvant, stimulated significantly higher neutralizing antibody titers, in both mice and non human primates, than those elicited by post-fusion forms.

We have recently explored the use of virus-like particles (VLPs) as vaccine candidates for RSV [22, 23]. Virus-like particles (VLPs) are now recognized as safe, effective vaccines for several viral diseases [24], notably those due to hepatitis B virus and human papilloma virus infections [24]. VLPs are virus-sized particles composed of repeating native structural arrays on their surfaces and in their cores, structures that mimic those of infectious viruses and contribute to their very potent immunogenicity [24–26]. We have recently developed novel VLPs containing RSV glycoproteins that induce, in mice, protective anti-RSV immune responses [22, 23]. These VLPs are based on the structural core proteins, nucleocapsid protein (NP) and matrix (M) protein, of Newcastle disease virus (NDV). To accomplish assembly of the RSV glycoproteins into these VLPs (ND VLPs), the ectodomains of the RSV F and G proteins were fused to the transmembrane (TM) and cytoplasmic tail (CT) sequences of the NDV fusion (F) and hemagglutinin-neuraminidase (HN) proteins, respectively, creating RSV F/NDV F (F/F) and NDV HN/RSV G (H/G) chimera proteins. VLPs containing these chimera proteins are highly immunogenic and stimulated both anti-RSV F protein and anti-RSV G protein specific antibodies in the absence of adjuvant [22, 23]. Recently, we reported the assembly, into these VLPs, of the ectodomain of the RSV F protein containing the mutations reported by McLellan et al. to stabilize the pre-fusion [21] or the post-fusion forms of this protein [17] and showed that, in mice and without adjuvant, the VLP associated pre-fusion F protein stimulated significantly higher titers of neutralizing antibodies than the VLP associated post-fusion F protein or wild type protein after a single immunization [27].

Cotton rats, which are more permissive to RSV infections than mice, are an accepted standard animal model for RSV [28]. Positive results in this model with vaccine candidates, anti-viral drugs, or antibodies have led directly to human trials. We previously immunized cotton rats with our wild type F containing VLPs and found that the resulting neutralizing antibody titers were unacceptably low (unpublished observations). Here we report results of immunization of cotton rats with the VLPs containing the pre-fusion RSV F protein (Pre-F/F VLPs) comparing responses to RSV infection. We found that Pre-F/F VLPs, without the use of adjuvant, stimulated high titers of neutralizing antibodies and that Pre-F/F VLP immunization of these animals protected them from virus replication in the lungs and in nasal passages upon RSV challenge. Furthermore, we report that Pre-F/F VLP immunization of cotton rats is safe. There was no pathology in these animals after immunization or after RSV challenge of immunized animals. Thus the pre-fusion F protein containing VLPs have characteristics required of a safe and effective RSV vaccine.

## Methods

### Cells, virus, plasmids

ELL-0 (avian fibroblasts), Vero cells, COS-7 cells, and HEp-2 cells were obtained from the American Type Culture Collection. All cells were grown in DMEM supplemented with penicillin, streptomycin, and containing 5 % (Vero cells) or 10 % fetal calf serum. RSV, A2 strain, was obtained from Dr. Robert Finberg.

The cloning and expression of the NDV NP and M protein have been previously described [29]. The construction, expression, and incorporation of the chimera protein NDV HN/RSV G (H/G) into VLPs have been previously described [23]. RSV G and RSV F sequences are from the A2 strain. Pre-fusion stabilized RSVF/NDV F (Pre-F/F) chimera protein was constructed as previously described [27]. Briefly, four point mutations, S155C, S190F, V207L, S290C, were introduced into the sequence encoding the ectodomain of the Gallus codon optimized RSV F protein (amino acid 1-524). This mutated F protein ectodomain sequence was fused to sequences encoding the Gallus codon optimized T4 fibritin trimerization motif (foldon) [21, 30], the RSV F protein sequence encoding amino acids 521–524 (the membrane proximal serine-threonine rich region common to paramyxovirus F proteins), followed by the sequences encoding the NDV transmembrane and cytoplasmic domains.

### Preparation of soluble forms of the pre-fusion and post-fusion F proteins and G protein

The soluble pre-F and post-F proteins were constructed as described by McLellan et al. [17, 21]. The soluble pre-F protein contained sequences encoding the RSV A2 F ectodomain (amino acids 1–513), containing the mutations S155C, S190F, V207L, S290C, fused to sequences (synthesized by GenWiz) encoding the foldon, the thrombin cleavage site, strep Tag II, GSGSG linker, and 6 × his tag [21]. The soluble post-fusion F protein was constructed using sequences encoding the RSV F protein ectodomain with the deletion of amino acids 137-146 fused to sequences encoding the thrombin cleavage site, strep Tag II, GSGSG linker, and 6x his tag [21].

To prepare the soluble G protein, the G protein gene sequence encoding the first 47 amino acids was deleted by PCR mutagenesis forcing initiation of translation at the methionine at amino acid 48. The truncated G protein was robustly expressed and secreted by COS-7 cells.

Soluble F proteins and soluble G protein were prepared from COS-7 cells, transfected with pCAGGS vectors containing sequences encoding the soluble pre-F protein, the soluble post-F protein, or soluble G protein, as previously described [27]. Supernatants harvested from cells transfected with pCAGGS DNA without an inserted gene were obtained for negative controls (CAGGS target).

### Antibodies

RSV F monoclonal antibody clone 131-2A (Chemicon) was used in RSV plaque assays. Monoclonal antibodies (mAb) 1112, 1200, and 1243, generous gifts of Dr. J. Beeler [31], and mAb 5C4, a generous gift of Dr. B. Graham [18], were used for ELISA analysis of VLPs and soluble F proteins [27]. Anti-RSV F HR2 peptide antibody [22] was used for quantification of soluble and VLP associated F proteins. Secondary antibodies specific for mouse and rabbit IgG were purchased from Sigma. Chicken anti-cotton rat IgG was purchased from Immunology Consultant Laboratories.

### Polyacrylamide gel electrophoresis, silver staining, and western analysis

For quantification of amounts of F and G proteins, VLPs or soluble F proteins were resolved on 8 % Bis–Tris gels (NuPage, Invitrogen). Silver staining of proteins in the polyacrylamide gels was accomplished as recommended by the manufacturer (Pierce). For Western blot analysis, proteins in the polyacrylamide gels were transferred to PVDF membranes using dry transfer (iblot, Invitrogen). Proteins were detected in the blots using anti-RSV HR2 peptide antibodies.

### VLP preparation, purification, and characterization

For preparations of VLPs to be used as immunogens, ELL-0 cells growing in T-150 flasks were transfected with cDNAs encoding the NDV M protein, NP, the chimeric protein H/G, and Pre-F/F as previously described [22, 23]. At 24 h post-transfection, heparin was added to the cells at a final concentration of 10 μg/ml [23] to inhibit rebinding of released VLPs to cells. At 48, 72, and 96 h post-transfection, cell supernatants were collected and VLPs were purified by sequential pelleting and sucrose gradient fractionation as previously described [22, 23, 32]. Concentrations of M, NP, and H/G proteins in the purified VLPs were determined by silver-stained polyacrylamide gels using marker proteins for standard curves [22, 32]. Determinations of amounts of F protein in VLPs or in soluble F protein preparations were accomplished by Western blots using anti-RSV F HR2 antibody for detection and comparing the signals obtained with a standard curve of purified F proteins as previously described [32].

### ELISA protocols

For determination of titers of IgG in sera of immunized animals by ELISA, wells of microtiter plates (Costar) were coated with cell supernatants containing soluble pre-fusion F protein (13 ng/well), soluble post-fusion F protein (13 ng/well), or soluble G protein (approximately 20 ng/well). Negative controls were equivalent volumes of supernatants from cells transfected with empty vector. Wells were then incubated in PBS-1 % BSA for 16 h. After washing wells three times with PBS, different concentrations of cotton rat sera were added to each well and incubated for 2 h at room temperature. After six washes in PBS, chicken anti-cotton rat antibody coupled to HRP was added in 50 μl PBS-1 % BSA and incubated for 1.5 h at room temperature. Bound HRP was detected by addition of 50 μl TMB (3,3′5,5′-tetramethylbenzidin, Sigma) and incubation for 5-20 min at room temperature until blue color developed. The reaction was stopped with 50 μl 1 N sulfuric acid. Color was read in SpectraMax Plus Plate Reader (Molecular Devices) using SoftMax Pro software. Antibody titers were defined as the log_10_ of the dilution that yielded an optical density of twice background. Reagents required to measure precise ng amounts of protein specific IgG in the cotton rat sera are not available.

### Preparation of RSV, RSV plaque assays, and antibody neutralization

RSV, strain A2, was propagated in HEp-2 cells after serial plaque-purification. The stock of virus utilized for the in vivo experiment, had a titer of 3.0 × 10^8^ pfu/ml. For virus titration, dilutions of virus or tissue samples were added to Hep-2 cells and incubated in 24-well plates at 37 °C after overlaying the wells with 0.75 % methylcellulose medium. After 4 days of incubation, the overlay was removed and the cells were fixed in 2.5 % glutaraldehyde solution containing 0.1 % crystal violet for 1 h, rinsed, and air-dried. Serum neutralization titers using RSV, strain A2, were determined as previously described [22, 23] and defined as the dilution of serum that reduced virus titer by 60 %.

### Animals, animal immunization, and RSV challenge

Cotton rats (*Sigmodon hispidus*) 4–6 weeks of age were immunized by intramuscular (IM) inoculation of 50 μg (low dose) or 150 μg (high dose) total VLP protein/animal (7.7 and 23.4 μg F protein, respectively) in 0.1 ml of TNE (50 mM Tris–HCl, pH 7.4, 150 mM NaCl, 5 mM EDTA). Boost immunizations of VLPs were 17 μg or 50 μg total VLP protein/animal (3.5 and 10.6 μg F protein, respectively). A group of control animals were immunized by intranasal inoculation with RSV, strain A2, (1 × 10^5^ pfu/animal). For initial infections, boosts, or challenges of animals with RSV, the animals were lightly anesthetized with isoflurane. All animal procedures and infections were performed in accordance with the Sigmovir IACUC and IBC approved protocols.

### Lung and nose viral titration

At 4 days after RSV challenge, five animals in each group were sacrificed and nasal tissue was harvested for virus titration. The lungs were excised en bloc and bisected for viral titration (left lobes) and histopathology (right lobes). Nasal tissue was obtained by dissection of the upper maxilla after skin removal. Viral titers in the lungs and in the nose of RSV-infected cotton rats were determined as previously described [33] and adjusted by the weight of the tissue portion. Briefly, lung and nose homogenates were clarified by centrifugation and diluted in EMEM. Confluent HEp-2 monolayers were infected in duplicate with diluted homogenates in 24 well plates. After 1 h incubation at 37 °C in a 5 % CO2 incubator, the wells were overlayed with 0.75 % methylcellulose medium. After 4 days of incubation, the overlay was removed and the cells were fixed with 0.1 % crystal violet stain for 1 h and then rinsed and air dried. Plaques were counted and virus titer was expressed as plaque forming units per gram of tissue. Viral titers were calculated as geometric mean ± standard error for all animals in a group at a given time.

### Pulmonary histopathology

The right lobes of lungs were dissected and inflated with 10 % neutral buffered formalin to their normal volume, and then immersed in the same fixative solution. Following fixation, the lungs were embedded in paraffin, sectioned and stained with hematoxylin and eosin (H&E). Four parameters of pulmonary inflammation were evaluated: peribronchiolitis (inflammatory cell infiltration around the bronchioles), perivasculitis (inflammatory cell infiltration around the small blood vessels), interstitial pneumonia (inflammatory cell infiltration and thickening of alveolar walls), and alveolitis (cells within the alveolar spaces). Slides were scored blind on a 0–4 severity scale [34].

### Statistical analysis

Statistical analyses (student T test) of data were performed using Graph Pad Prism 7 software.

## Results

### Properties of VLPs containing a pre-fusion RSV F protein

As previously described [27], the stabilized pre-fusion form of the RSV F protein (Pre-F/F) was incorporated into NDV VLPs by introducing into the previously characterized wild type F/F chimera protein [22] four point mutations in the RSV F protein ectodomain and adding to the carboxyl terminus of the RSV F protein ectodomain a trimerization domain, the “foldon” sequence, as these were changes identified by McLellan et al. [21] to be necessary for the stabilization of the secreted form of the pre-fusion F protein trimer. In addition to the Pre-F/F protein, these VLPs contained the H/G chimera protein and the NDV NP and M proteins. Verification of the conformation of the Pre-F/F protein in VLPs [27] was accomplished using monoclonal antibody 5C4, which binds site ϕ specific to the pre-fusion form of the RSV F protein [18]. In addition, the Pre-F/F VLPs bound monoclonal antibodies [27] specific to each of the previously defined F protein antigenic sites I, II, and IV [31] indicating that the Pre-F/F containing VLP also contains those sites as previously reported for soluble forms of the pre-fusion F protein [17, 18].

### Anti-F and anti-G protein IgG immune responses to the VLPs

To assess the immunogenicity of the Pre-F/F VLPs (VLP-H/G + Pre-F/F) in cotton rats, groups of ten cotton rats were immunized, by intramuscular injection (IM) with a low dose or a high dose of VLP-H/G + Pre-F/F or sham immunized with buffer. Other groups of 10 cotton rats were infected, intranasally (IN), with infectious RSV or not immunized. For assessment of the safety of VLP immunization, discussed below, a group of ten cotton rats was immunized IM with formaldehyde inactivated RSV (FI-RSV). At day 21 post prime immunization, all groups of cotton rats were boosted with buffer, VLPs, or FI-RSV by IM inoculation or with RSV by IN inoculation. Sera were harvested by periorbital bleeding at 14, 21, 35, and 49 days. Aliquots of sera obtained from the ten animals at each time point were pooled for determination of anti-F protein and anti-G protein IgG responses with time after immunization. Anti-F protein IgG responses of individual animals at day 49 were also determined.

**Fig. 1:**
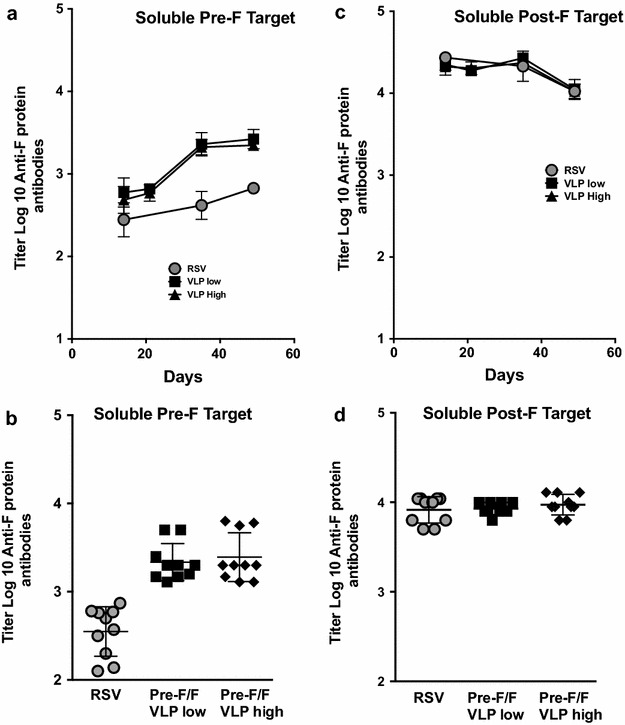
Anti-RSV F protein IgG immune responses to soluble Pre-F and Post-F target antigens. Figure shows titers with time after immunization of anti-F protein IgG in pooled sera that bind to the soluble pre-fusion F protein (**a**) and that bind to the soluble post-fusion F protein (**c**). Data shown are the averages of four separate determinations. **b** and **d** show titers in sera from individual cotton rats obtained at 49 days post immunization that bind to soluble pre-fusion F protein (**b**) or that bind to soluble post-fusion F protein (**d**). **b** Differences between the RSV group and the VLP groups are significant (p ≤ 0.0001) while the differences between VLPs low and VLPs high are not significant. **d** Differences between groups are not significant. Titers are defined in “Methods”. Pre-F/F VLPs low and Pre-F/F VLPs high indicate a dose (IM) of 50 and 150 µg total VLP protein/animal, respectively in the prime immunization. Boost immunizations, at day 21, were 17 and 50 μg/animal, respectively. RSV prime and RSV boosts were 1 × 10^5^ pfu/animal (IN)

To characterize the binding of anti-F protein antibodies induced by the Pre-F/F VLPs to the pre-fusion and post-fusion forms of the F protein, we prepared soluble versions of both pre-F and post-F proteins to use as targets in ELISA as previously described [21, 35]. We have previously reported verification of the conformation of these soluble forms of F protein by the binding of representative anti-F monoclonal antibodies to these secreted proteins in ELISA assays [27]. The binding of antibodies in cotton rat sera after VLP immunization or RSV infection to the soluble pre-fusion and post-fusion F proteins by ELISA is shown in Fig. 1.

Using soluble pre-fusion F protein as target, the day 14 sera from VLP-H/G + Pre-F/F immunized animals contained slightly higher titers of F protein specific IgG than sera from RSV infected cotton rats. However, after boosting, binding to pre-fusion F protein increased significantly in VLP-H/G + Pre-F/F immunized animals relative to those in sera obtained from RSV immunized animals (Fig. 1a, b). The dose of VLPs did not have a significant effect on titers of pre-fusion F protein specific IgG antibodies. The variability between VLP immunized animals at day 49 was minimal (panel b). Thus the Pre-F containing VLPs were better at stimulating antibodies that would bind to the soluble pre-F protein than RSV infection.

Using the soluble post-fusion F protein as target, the titers of IgG in sera from VLP-H/G + Pre-F/F immunized animals were similar to those stimulated by RSV infection (Fig. 1c, d). The variability between animals at day 49 was minimal (panel d). Boosting with VLPs or RSV had little effect on titers of post-F specific IgG. Different amounts of VLP immunogen had no effect on titers.

**Fig. 2:**
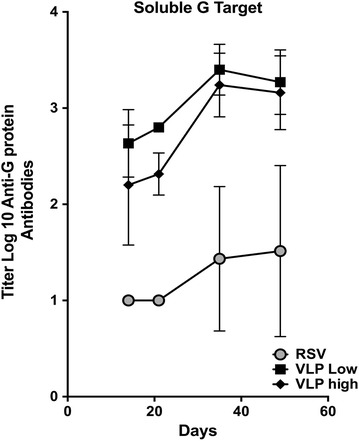
Anti-RSV G protein IgG immune responses to soluble RSV G protein. Figure shows titers with time, after immunization with VLP-H/G + Pre-F/F or RSV, of anti-G protein IgG in pooled sera that bind to the soluble G protein. Data are the average of three separate determinations. VLP and RSV immunizations were as in Legend to Fig. 1. Boost immunization was at day 21

The levels of anti-G antibody responses after immunization in the VLP immunized animals and in RSV infected animals were compared in an ELISA using soluble RSV G protein as target antigen. Figure 2 shows the titers of anti-G protein antibodies in pooled sera with time after immunization. Clearly VLP immunization stimulated significantly higher levels of anti-G protein IgG responses than RSV infection. The differences between levels of anti-G protein antibodies in sera from high dose and low dose VLPs were not statistically significant.

### Neutralizing antibody responses stimulated by the VLPs

**Fig. 3:**
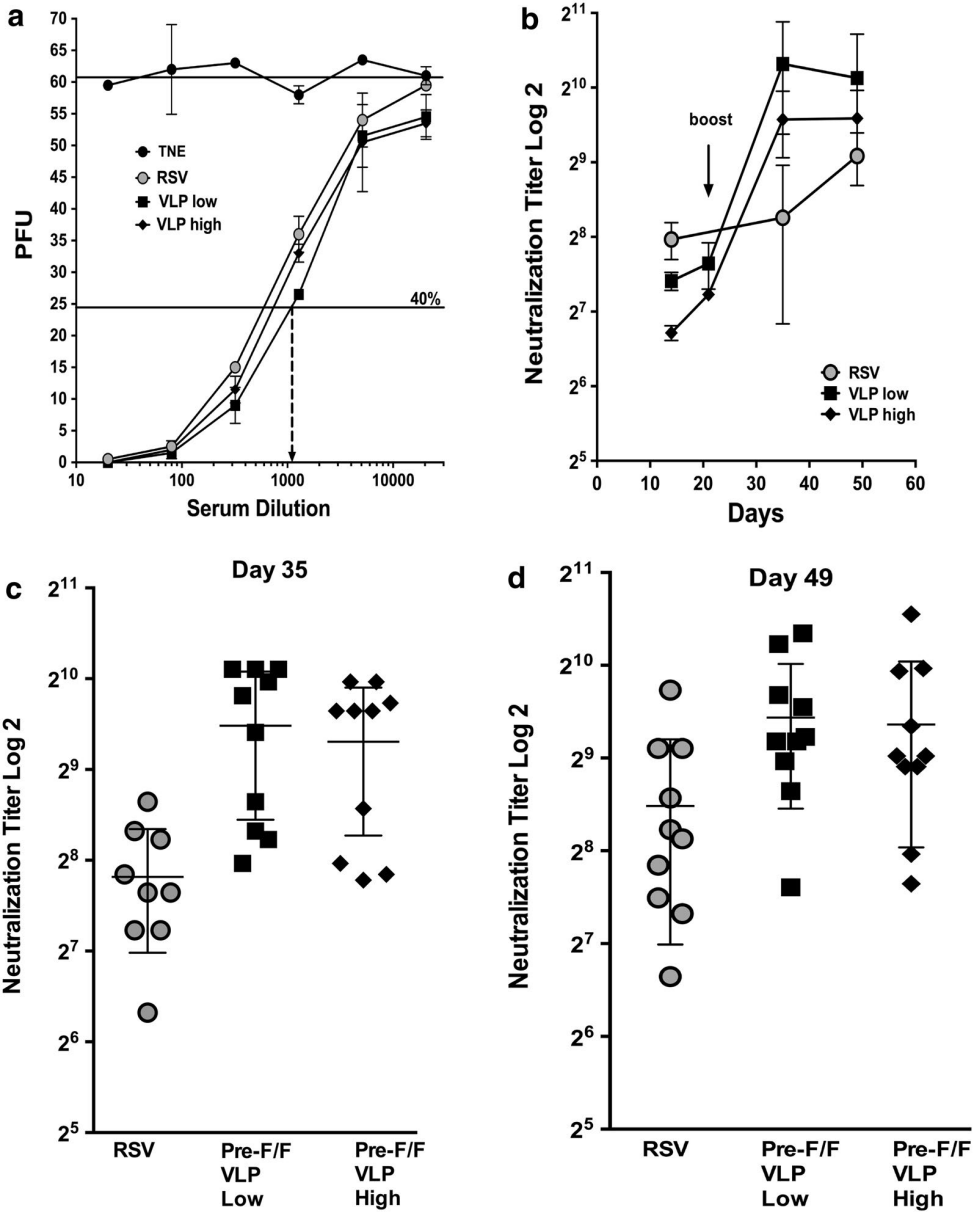
Virus neutralization titers in sera from immunized cotton rats. **a** Representative data from plaque reduction assays of pooled sera obtained at 49 days post immunization and used to determine neutralization titers. *Arrow* indicates titer for VLP-low dose. **b** RSV A2 neutralization titers with time in pooled sera. Data are the average of five separate determinations by the method shown in **a**. *Arrow* indicates time of the boost immunization. RSV A2 neutralization titers in sera from individual cotton rats at day 35 (**c**) and day 49 (**d**) post immunization, determined as shown in **a**. Immunogens are shown at the bottom of the panels. Differences between the RSV group and the VLP groups are significant (p = 0.0012 and p = 0.019, respectively). Differences between the two VLP groups are not significant

Methods to determine neutralization titers vary from report to report. We chose to determine the neutralizing antibody titer of sera in a classical in vitro plaque reduction assay. For these assays, pooled sera were used as were sera from individual cotton rats. For kinetics of induction of neutralizing antibodies, equal aliquots of sera from animals in each group obtained at four times after immunization were pooled and used to determine titers. Figure 3, panel a, shows an example of data used to determine these neutralization titers. Values obtained using buffer immunized animals (TNE) were taken as 100 % to account for any nonspecific neutralization by pre-immune sera. Curves show plaques obtained after incubation of virus with increasing amounts of sera from buffer, RSV or VLP immunized animals. The dilution of sera resulting in reduction in titer by 60 % of the control was used to determine neutralization titers (indicated by the arrow for VLP low dose sera). Panel B shows neutralization titers with time after immunization determined in this way in pooled sera. Similarly, the titers of sera from each cotton rat were determined in sera harvested at day 35 and day 49 (panels c and d). Figure 3 shows that after the prime immunization at day 14, RSV infection stimulated slightly higher titers of neutralizing antibodies than VLP-H/G + Pre-F/F immunization. However, the VLPs stimulated significantly higher titers of neutralizing antibodies than RSV infection after the boost immunization, a result obtained with pooled sera (panel b) as well as sera from individual animals (panels c and d). While titers were higher on average at day 35 than day 49, the difference was not statistically significant. The variability in neutralizing titers between individual animals is shown in panels C and D.

The titers obtained after immunization of cotton rats with the wild type F containing VLPs (F/F) were 4–5 log_2_, at day 43 (unpublished observations), which is approximately 22 times lower than those obtained after immunization with the pre-F containing VLPs. Thus the Pre-F containing VLPs are a much more effective immunogen than the wild type F containing VLPs and approximately three times more effective than RSV infection.

### Protection of immunized animals from RSV challenge

**Fig. 4:**
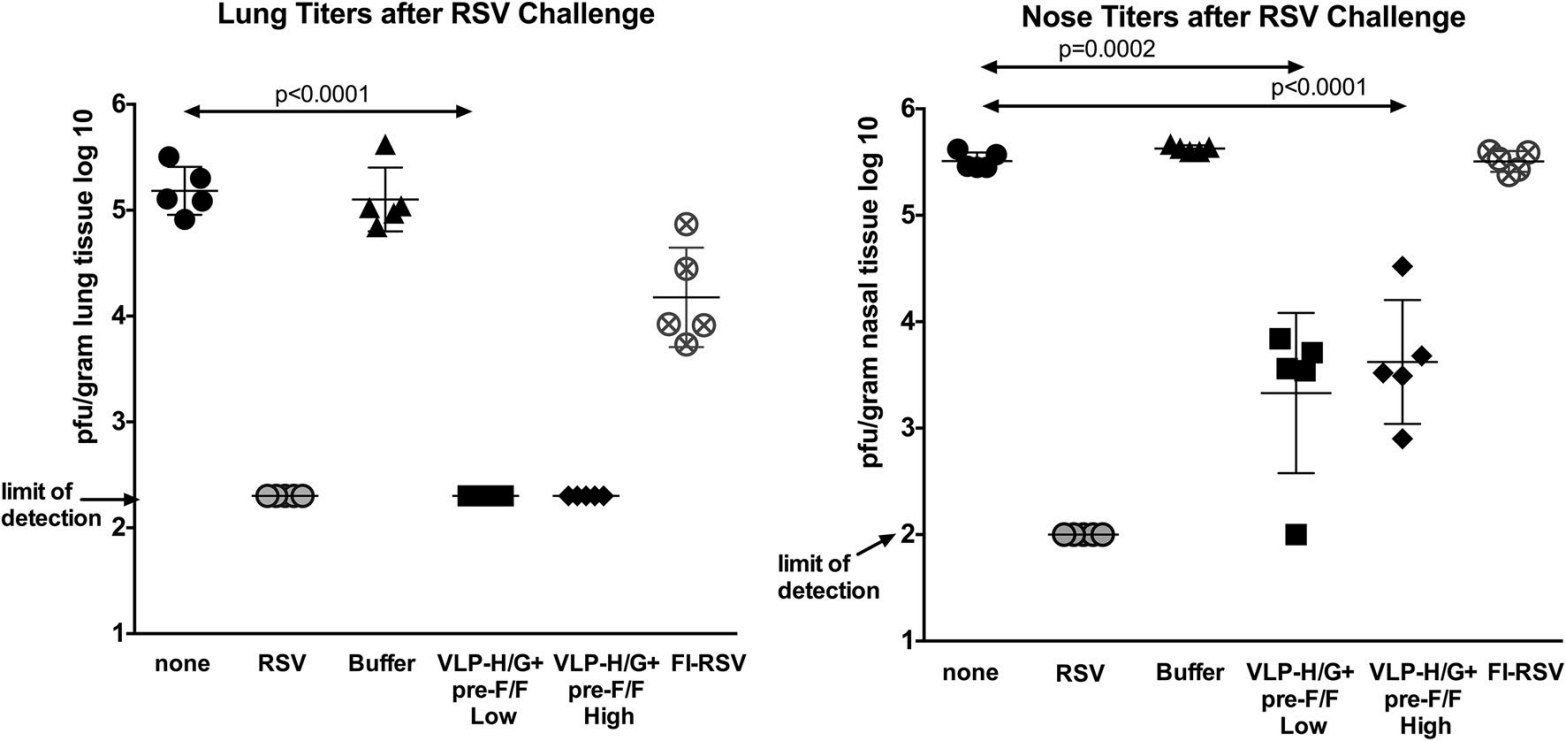
Protection of immunized cotton rats from RSV challenge. *Panels* show titers of virus/gm in lung (*left*) and nasal (*right*) tissue four days after RSV A2 challenge of five cotton rats at 49 days post immunization. Immunogens are shown at the bottom for each group of five animals. Statistically significant differences between groups are indicated by p values at the top of each *panel*

To determine the protection from RSV replication afforded by VLP-H/G + Pre-F/F immunization, the virus titers in lungs and in nasal passages of immunized rats after RSV challenge were determined by plaque assay (Fig. 4). Clearly VLP-H/G + Pre-F/F immunization protected mice from RSV replication upon challenge as did previous RSV infection. VLP immunization completely protected the animals from replication in the lungs. VLP immunization reduced the titer of RSV in the nasal tissue by two logs.

### Lung pathology in immunized animals after RSV challenge

**Fig. 5:**
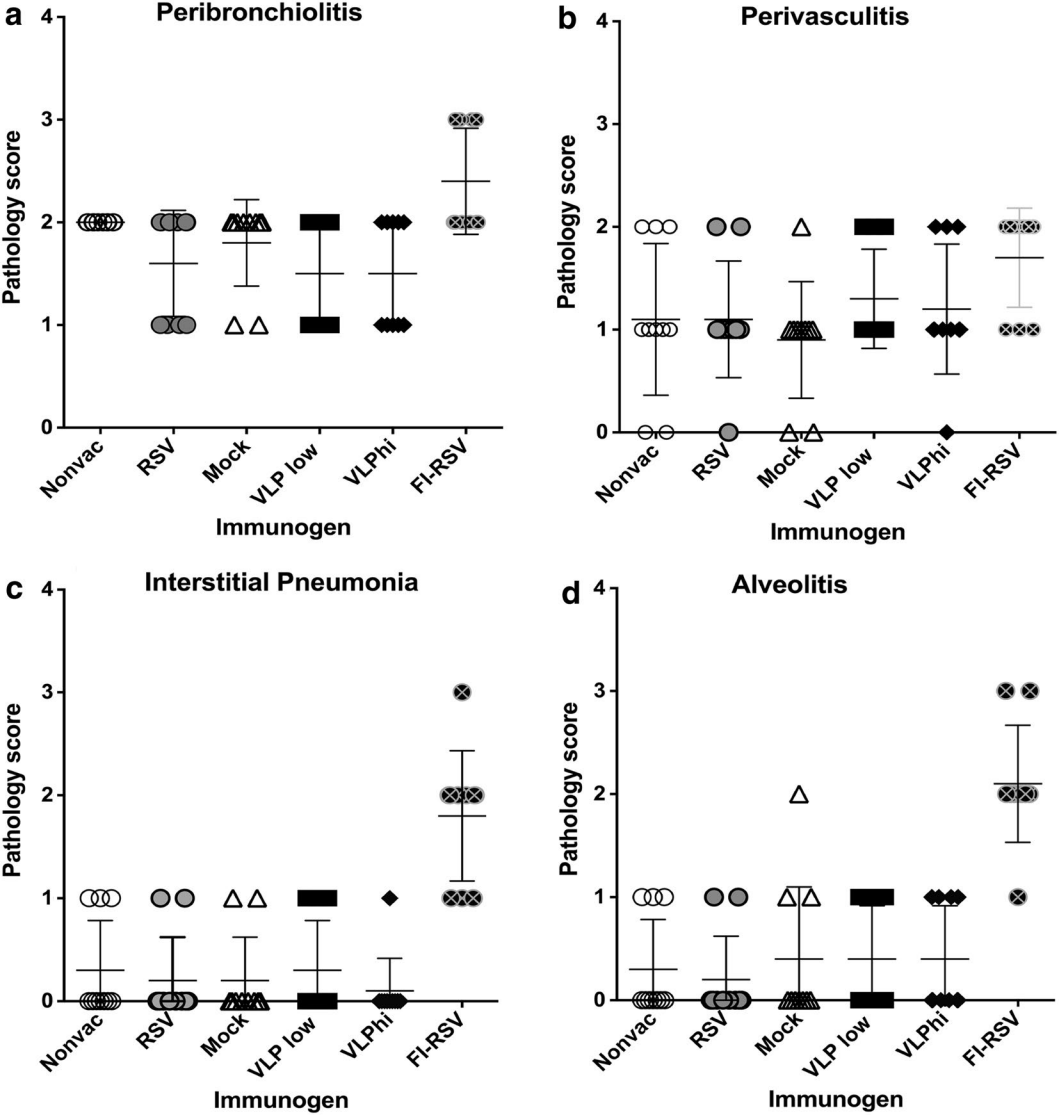
Assessment of lung pathology after RSV challenge of immunized cotton rats. *Panels* show scores of lung inflammation in ten cotton rats/group immunized with immunogens indicated at the *bottom of each panel* and then challenged with RSV at 49 days post immunization. At 4–5 days post challenge, lungs were harvested and tissue sections stained and scored for inflammation as described in "Methods". Differences between FI-RSV immunized animals and all other groups are statistically significant except for scores of perivasculitis, which are not significant. Differences between mock vaccinated, RSV infected, and VLP vaccinated animals in all categories are not statistically significant.

**Fig. 6:**
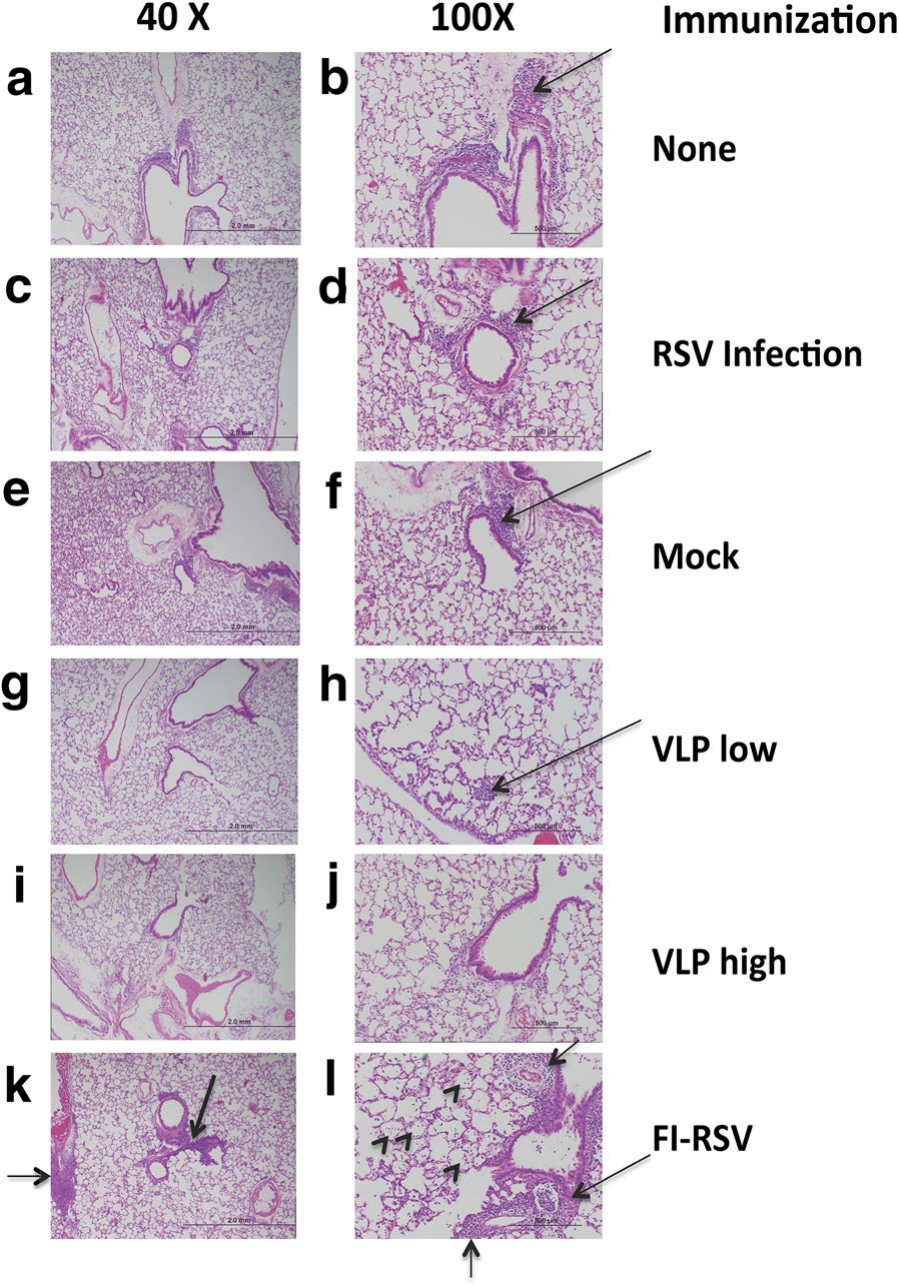
Lung sections of RSV challenged immunized cotton rats. *Panels* show representative H&E stained lung sections of cotton rats that were not immunized (**a**, **b**), immunized with RSV (**c**, **d**), mock immunized (**e**, **f**), immunized with low dose Pre-F/F containing VLPs (**g**, **h**), or high dose Pre-F/F containing VLPs (**i**, **j**), or FI-RSV immunized animals (**k**, **l**), all after an RSV challenge at 45 days post immunization. *Large arrows* indicate patches of inflammatory cells. *Small arrowheads* indicate cell infiltration into the alveolar space (alveolitis). **a**, **c**, **e**, **g**, **i**, **k** show ×40 magnification while **b**, **d**, **f**, **h**, **j**, **l** show ×100 magnification

We have previously reported that RSV challenge of VLP-H/G + F/F immunized mice did not result in lung pathology typical of that seen after RSV challenge of mice immunized with formalin inactivated RSV (FI-RSV) [22]. The cotton rat, which is more permissive for RSV than BALB/c mice, is a better animal model than mice for assessment of enhanced respiratory disease following RSV challenge of an RSV vaccine candidate recipient. Thus the safety of immunization with Pre-F/F containing VLPs was assessed in this animal model. Five groups of cotton rats were immunized with two concentrations of VLP-H/G + Pre-F/F, sham immunized with buffer, infected with RSV, or not immunized. A sixth group was immunized with FI-RSV and served as the positive control for enhanced respiratory disease. All groups of animals were challenged, IN, with infectious RSV. At 4–5 days following the challenge, lungs were harvested for histological analysis of inflammation and lung sections were scored blindly for peribronchiolitis, perivasculitis, interstitial pneumonia, and alveolitis. The scores are shown in Fig. 5, panels a–d, respectively. Immunization with FI-RSV recapitulated previously reported abnormal histology of the lung sections. The lungs of VLP-H/G + Pre-F/F immunized cotton rats had scores that were statistically significantly lower than scores for FI-RSV immunized cotton rats and comparable to those of sham vaccinated or previously RSV infected animals. Figure 6 shows representative pictures of the H&E stained lung sections. Arrows indicate of inflammatory cell infiltration in these lungs, which is significantly increased in lungs of FI-RSV immunized animals (panels k and l) compared to that in lungs of VLP immunized animals (panels g–j), RSV infected (panels c and d), or mock immunized animals (panels e and f). Arrowheads indicate alveolitis. Thickening of airway walls was also particularly enhanced in lungs of FI-RSV immunized animals (panel l). These results indicate that VLP vaccination of cotton rats does not stimulate enhanced respiratory disease, results similar to our previous reports of VLP immunization of mice.

## Discussion

Numerous RSV vaccine candidates have been tested in animals and in human trials but no vaccine candidate has been licensed because of failure of these vaccine candidates to provide significant protection from RSV infection in humans [9, 10]. It is quite likely that all non-replicating vaccine candidates, such as soluble protein, have contained predominantly the post fusion form of the F protein, which could account for failure of these candidates. Furthermore, a recent report indicates that the RSV F protein in virus particles exists largely in the post-fusion form suggesting the hypothesis that attenuated RSV vaccine candidates or even wild type RSV infections present predominately the post-fusion F protein to the immune system [36]. As shown by Magro et al., most neutralizing antibodies in human or rabbit sera do not bind to the post-fusion F protein [20] and, as shown by McLellan et al., monoclonal antibodies that will bind the post-fusion F require far higher concentrations to neutralize RSV than antibodies specific to the pre-fusion F protein [18]. Indeed, we have previously reported that RSV infection of mice induces approximately a ten to 50-fold higher concentrations of IgG that bind to soluble post-fusion F targets than IgG that binds to soluble pre-fusion F targets [27]. Such a phenomenon could account, in part, for the failure of infectious vaccine candidates or wild type RSV to provide protection from subsequent infections.

McLellan et al. reported mutations in the RSV F protein that stabilize the pre-fusion F protein [21]. We have incorporated these changes into the RSV F/NDV F (F/F) chimera protein in our ND VLPs and demonstrated that the Pre-fusion F/F containing VLPs induced, in BALB/c mice, significantly higher neutralization titers than VLPs constructed to contain a stabilized post-fusion F protein (Post-F/F) and significantly higher neutralization titers than RSV infection [27]. Because the Post-F/F containing VLPs resulted in lower neutralization titers than the Pre-F/F containing VLPs in mice, we chose to extend these studies by characterizing immune responses only to the Pre-F/F containing VLPs in cotton rats, a preferred animal model for testing of RSV vaccine candidates [28]. We did compare responses to the Pre-F/F containing VLPs to RSV infection in these animals. Cotton rats are more permissive to RSV infection than BALB/c mice. Thus RSV infection should stimulate better immune responses in cotton rats than in mice and provide a more stringent test of the effectiveness of the VLP immunization and the safety of the vaccine candidate in immunized animals.

The most important results presented here are neutralization titers in cotton rats after VLP immunization. There have been, over the years, various estimates of the minimum serum neutralization titer required for protection from RSV challenge. Based on serum protective levels of the antibody palivizumab in infants, a minimal titer was defined as greater than or equal to 100 (reciprocal of dilution of sera resulting in 50 % reduction in RSV/A2 virus titer in a plaque reduction assay) [21]. Another estimate, based on cotton rat studies, suggested titers of greater than 6, log 2, (60 % reduction in RSV/A2 virus titer) [37]. Other investigators have specified titers of 380 or 390 (reciprocal of serum dilutions) [38] or 8.5 log_2_ (60 % reduction) [39]. Our results showed that Pre-F/F containing VLPs, even after a single or prime immunization, stimulated serum titers of 7.5 log_2_ (60 % reduction in titer) and a boost immunization stimulated the titers, on average, of between 9 and 10 (60 % inhibition). Thus these titers are considerably above all the threshold levels deemed necessary for a successful vaccine candidate. Significantly, the VLPs stimulated better neutralizing antibody titers than RSV infection after a boost. This result demonstrates the effectiveness of the VLP as an immunogen in spite of the fact that this particle is a non-replicating antigen in contrast to the RSV infection.

VLPs used for these experiments contained not only the Pre-F/F but also the RSV G protein ectodomain since antibodies specific to the G protein have been shown to be protective in vivo [40–42]. Serum neutralization titers measured here in an in vitro plaque reduction assay could be due to antibodies to both the RSV F and G protein. However, we have constructed a VLP containing only the H/G chimera protein and reported that the neutralization titers induced by this VLP in mice were extremely low, titers of approximately 3 log_2_ [23]. It is unlikely that the combination of G and F in a VLP will increase neutralization titers attributable to the G protein in these in vitro assays. However, the presence of G protein sequences in these VLPs likely has an important role in stimulation of protection from RSV replication and absence of increased lung inflammation upon RSV challenge. G protein has been associated with suppression of anti-RSV immune responses [43] and stimulation of enhanced respiratory disease [44]. Antibodies to G protein are known to decrease virus replication in lungs upon infection [45] and can ameliorate lung pathology after RSV challenge of FI-RSV immunized animals [46].

Consistent with the stimulation of robust neutralizing antibody titers, the VLP immunization induced protective responses in cotton rats. An RSV challenge of VLP immunized animals completely protected them from replication in lungs. While some virus was detected in nasal passages upon RSV challenge of these animals, titers were reduced by 2–2.5 log_10_ over the unimmunized animals. RSV challenge of RSV infected cotton rats reduced nasal titers to undetectable levels. Perhaps this better protection from nasal passage replication by previous RSV infection compared to VLP immunization is related to the differing routes of immunization, intranasal vs intramuscular. Accordingly, intranasal immunization with the VLPs may stimulate better mucosal immunity and induce upper respiratory tract protection at levels comparable to those induced by previous RSV infection, a possibility to be explored in future experiments.

We have previously reported that, in mice, VLP-H/G + Pre-F/F immunization and RSV infection stimulated equivalent titers of IgG that bound to a soluble form of the post-fusion F protein in an ELISA [27]. Here we report similar results in cotton rats. However, as we found in mice [27], VLP immunization of cotton rats stimulated significantly higher titers of IgG that bound to pre-fusion F protein than RSV infection, a result suggesting that the VLP is more effective at presenting pre-fusion F specific epitopes to the cotton rat immune system than RSV infection.

In contrast to results in the murine system [27], a boost immunization of VLPs or RSV did not increase titers to the post-fusion F protein while titers in mice significantly increased upon a boost. While a second RSV infection (boost) only slightly increased titers to the pre-fusion F protein, VLP immunization significantly increased titers to the pre-fusion F protein, similar to results in mice and mirroring increases in neutralization titers. These results may indicate that the nature or induction of immune responses specific to the post-F protein in cotton rats are different from mice and different for antibodies to pre- and post F targets in cotton rats. Alternatively, the time interval between the prime and boost immunization in the cotton rats may have been too short to detect a boost of post-fusion F specific serum antibody titers. In contrast to pre-fusion F specific titers, the higher antibody concentrations to the post-F protein after the prime in cotton rats may well have interfered with further stimulation of antibodies specific to the soluble post-fusion F protein.

A very important property of any RSV vaccine candidate that must be addressed is safety, particularly for non-replicating vaccines. This issue has dominated RSV vaccine development for years because an early vaccine candidate, a formalin-inactivated preparation of purified virus (FI-RSV), not only failed to protect infants from infection, but also unexpectedly resulted in enhanced, life-threatening respiratory disease (ERD) upon subsequent infection with RSV (reviewed in [47–50]). We have reported that immunization of mice with three different versions of RSV F containing VLPs did not stimulate enhanced respiratory disease upon RSV challenge [22, 23, 51] even at late times after immunization, in contrast to some other non-replicating RSV vaccine candidates [52, 53]. Here, we showed, in cotton rats, that RSV challenge after VLP-H/G + Pre-F/F immunization did not result in lung pathology while the positive control for ERD, FI-RSV, recapitulated the lung pathology repeatedly observed with this immunogen. Thus our results support the conclusion that immunizations with VLPs do not result in enhanced respiratory disease upon subsequent RSV infection, in contrast to some other non-replicating immunogens [52, 53].

## Conclusions

Our results show that VLPs based on the core proteins of Newcastle disease virus M and NP and expressing on VLP surfaces the stabilized pre-fusion form of the RSV F protein as well as the RSV G protein induced in cotton rats robust neutralizing antibody responses and protective responses. Importantly, our results show that this vaccine candidate does not induce enhanced respiratory disease upon RSV challenge, in contrast to responses to some non-replicating vaccine candidates [53, 54]. In addition, these VLPs should be particularly safe since they are non-replicating and incapable of a spreading infection characteristic of infectious virus vectored RSV vaccine candidates.

## Abbreviations

VLPs: virus-like particles
RSV: respiratory syncytial virus
ELISA: enzyme-linked immunsorbant assay
pfu: plaque forming units
F: fusion protein
G: glycoprotein
HN: hemagglutinin-neuraminidase protein
IgG: immunoglobulin G
F/F: chimera protein containing the ectodomain of the RSV F protein and the transmembrane and cytoplasmic tail of the Newcastle disease virus F protein
H/G: chimera protein containing the ectodomain of the RSV G protein and the transmembrane and cytoplasmic tail domain of the Newcastle disease virus HN protein
NDV: Newcastle disease virus
M: membrane protein
NP: nucleocapsid protein
HRP: horseradish peroxidase
PBS: phosphate buffered saline
TNE: tris, NaCl, EDTA containing buffer
TM: transmembrane domain
CT: cytoplasmic domain
